# ^11^C-acetate PET/CT in pre-therapeutic lymph node staging in high-risk prostate cancer patients and its influence on disease management - a retrospective study

**DOI:** 10.1186/s13550-014-0055-1

**Published:** 2014-10-09

**Authors:** Sara Strandberg, Camilla Thellenberg Karlsson, Torbjörn Sundström, Mattias Ögren, Margareta Ögren, Jan Axelsson, Katrine Riklund

**Affiliations:** Department of Radiation Sciences, Umeå University, 90185, Umeå, Sweden

**Keywords:** Prostatic neoplasms, PET/CT, ^11^C-acetate, Neoplasm staging, Lymphatic metastasis

## Abstract

**Background:**

Radiation treatment with simultaneous integrated boost against suspected lymph node metastases may be a curative therapeutic option in patients with high-risk prostate cancer (>15% estimated risk of pelvic lymph node metastases according to the Cagiannos nomogram). ^11^C-acetate positron emission tomography/computed tomography (PET/CT) can be used for primary staging as well as for detection of suspected relapse of prostate cancer. The aims of this study were to evaluate the association between positive ^11^C-acetate PET/CT findings and the estimated risk of pelvic lymph node metastases and to assess the impact of ^11^C-acetate PET/CT on patient management in high-risk prostate cancer patients.

**Methods:**

Fifty consecutive prostate cancer patients referred for primary staging with ^11^C-acetate PET/CT prior to radiotherapy with curative intention were enrolled in this retrospective study.

**Results:**

All patients showed increased ^11^C-acetate uptake in the prostate. Pelvic lymph node uptake was seen in 42% (21/50) of the patients, with positive external iliac lymph nodes in 71% (15/21) of these. The overall observed proportion of PET/CT-positive pelvic lymph nodes at patient level was higher than the average estimated risk, especially in low-risk groups (<15%). There was a significant association between observed proportion and estimated risk of pelvic lymph node metastases in groups with ≤45 and >45% estimated risk. Treatment strategy was altered due to ^11^C-acetate PET/CT findings in 43% (20/47) of the patients.

**Conclusions:**

The observed proportion of ^11^C-acetate PET/CT findings suggestive of locoregional metastases was higher than the estimated risk, suggesting that the Cagiannos nomogram underestimates the risk for metastases. The imaging results with ^11^C-acetate PET/CT have a considerable impact on patient management.

## Background

During a lifetime, approximately 15% of men will be diagnosed with prostate cancer, making it the second most common cancer form in males worldwide [1]. According to the Swedish National Prostate Cancer Registry, it is the most common cancer in males in Sweden, with an incidence of approximately 10,000 and a mortality of 2,500 annually [2]. Prostate cancer is a complex heterogeneous disease with highly variable morphological and physiological characteristics [3]. Staging and risk categorisation is decided from clinical features including T stage, prostate-specific antigen (PSA) and Gleason score. Risk estimation can be facilitated with the use of nomograms such as the Cagiannos nomogram, which is a widely used clinical tool to determine the extent of disease as well as the risk of pelvic lymph node involvement [4]. Prostate cancer with >15% estimated risk of pelvic lymph node metastases is considered as high-risk prostate cancer but still stands a chance to get cured with standard radiotherapy including the prostate and the seminal vesicles (78 Gy) [5]. However, a more aggressive treatment approach including the pelvic lymph nodes is under development [5] and is used in the treatment of high-risk prostate cancer in the University Hospital of Umeå. Nomograms will only provide an estimated risk of pelvic lymph node metastasis, and it is necessary to establish a more reliable non-invasive method for staging. Hybrid imaging with positron emission tomography/computed tomography (PET/CT) renders various molecular and morphological information, depending on the radiotracer and the design of the CT protocol. PET/CT is widely used in oncologic imaging for cancer staging and restaging as well as for evaluation of treatment response. The most used radiotracer ^18^F-FDG (2-deoxy-2-[^18^F]-d-glucose) is an indicator of glucose metabolism, which has proved useful in several malignant tumours due to their increased glycolysis, the Warburg effect [6], but it has limitations in relatively slow-growing malignancies such as prostate cancer. For PET/CT imaging in prostate cancer, ^11^C-acetate, ^11^C-choline, and ^18^F-choline appear to be more suitable tracers [7]. Multiple other promising new tracers are under development for imaging in prostate cancer but still need further investigation [8,9]. Currently, ^11^C-/^18^F-choline and ^11^C-acetate are the most commonly used tracers, with comparable results [7,10]. A recent study by Buchegger et al. showed excellent concordance between ^18^F-choline and ^11^C-acetate in the detection and localization of positive lymph nodes and skeletal metastases in patients with prostate cancer [10]. ^11^C-choline-PET/CT has proved valuable mainly for radiotherapy planning in prostate cancer patients with biochemical recurrence after radical treatment [11]. ^11^C-acetate is a radiotracer that is believed to reflect lipid metabolism, but its exact pharmacodynamic mechanism is still debated [12]. Several studies indicate that ^11^C-acetate is of value for primary staging of prostate cancer [8,13–15]. Oyama et al. have shown ^11^C-acetate to be superior to ^18^F-FDG with higher sensitivity for prostate cancer and its metastases [13]. In 2012, Castellucci and Jadvar suggested in a review of PET/CT in prostate cancer that the main application for ^11^C-acetate might be to rule out distant metastases in early prostate cancer relapse prior to salvage radiotherapy [8].

The aims of this retrospective study were to evaluate the correlation between ^11^C-acetate PET/CT findings and the estimated risk of locoregional lymph node metastases as indicated by the Cagiannos nomogram and to assess the impact of ^11^C-acetate PET/CT examination results on the clinical management of previously untreated high-risk prostate cancer patients.

## Methods

### Patients and clinical data

Fifty consecutive patients referred for ^11^C-acetate PET/CT at Nuclear Medicine, Department of Radiology, University Hospital of Umeå, Sweden, from 2011 July 6 to 2013 March 26 were included. The cause for referral was primary staging of biopsy-verified prostate cancer prior to radiotherapy with curative intention. Patients were referred for ^11^C-acetate PET/CT if they reached 15% risk in the nomogram or if they exhibited other risk factors such as rapidly increasing PSA. The time interval between biopsy and ^11^C-acetate PET/CT was at an average of 8 weeks, range 3 to 18 weeks. All patients had a baseline ^11^C-acetate PET/CT scan, of which 90% (45/50) had a contrast-enhanced CT (CECT) scan in the same session. The remaining five patients had a diagnostic CT scan without intravenous (i.v.) contrast due to either impaired renal function or a previous examination with i.v. iodine contrast media within 4 weeks.

Clinical information regarding pre-treatment status was retrieved from the referral text. Follow-up data were collected from the patients' medical records. Clinical data concerning changes in treatment strategy were missing in three patients referred from regional hospitals, and these patients were excluded from the evaluation of impact of ^11^C-acetate PET/CT findings on treatment strategy. The study was approved by the regional ethics review board.

The Cagiannos pre-treatment nomogram was used to estimate the risk of pelvic lymph node involvement. Factors taken into account in the nomogram are age, pre-treatment PSA, clinical tumour stage, primary and secondary Gleason grade, and the number of positive and negative biopsy cores [16]. Estimated risk data could be calculated for all patients except one, where clinical data were missing. This patient was excluded from the evaluation of the correlation between ^11^C-acetate PET/CT findings and the estimated risk of locoregional lymph node metastases. Patient characteristics are shown in detail in Table 1.

**Table 1:** **Patient characteristics**

	***n***	**Mean**	**Range**
Patients (*n*)	50		
Age (years)		67	41 to 77
PSA (ng/ml)		37	2.7 to 168
Gleason score		8	6 to 10
Estimated risk of locoregional lymph node metastases (%)^a^		32	3.3 to 80.3

^a^According to the Cagiannos pre-treatment nomogram, where age, pre-treatment PSA, clinical tumour stage, primary and secondary Gleason grade, and the number of positive and negative biopsy cores influence the estimated risk.

### PET/CT imaging

All image acquisition was done with a GE Discovery 690 PET/CT scanner (General Electric, Pewaukee, WI, USA). Software and algorithms were supplied with the scanner. The patients were injected with 1-[^11^C]-acetate (5.0 MBq/kg body weight, mean dose 436 MBq, range 304 to 577 MBq) i.v., and a CT scan with low dose for PET attenuation was acquired, followed by a PET scan 10 min post-injection and finally a diagnostic CT with or without i.v. contrast media. The attenuation CT was a helical 0.5-s rotation time scan, employing 120 kV and 30 mA. The PET scan was performed in time-of-flight mode with an acquisition time of 2 min/bed position, including the abdomen, thorax and neck. The PET images were reconstructed with the OSEM-based VuePoint HD (GE Healthcare, Pewaukee, WI, USA) (2 iterations, 24 subsets, 6.4 mm Gaussian filter), to a 128 × 128 pixel matrix with 50-cm field-of-view, giving a voxel size of 5.5 × 5.5 × 3.27 mm^3^. The diagnostic CT included the neck, thorax and abdomen, using 120 kV, with beam current controlled by the Auto-mA algorithm (noise index 35, current limited to the range 150 to 750 mA). The CECT was done after i.v. injection of iodine contrast (Omnipaque 350 mgI/ml 0.5 g I/kg, rendering a patient mean volume of 137 ml, range 108 to 167 ml).

### Evaluation of image data

Two physicians double licensed in radiology and nuclear medicine (one with >10 years' experience from reading PET/CT and one with 2 years' experience) visually evaluated all PET/CT studies. Any exceptional cases of inter-observer disagreement were solved with consensus. The radiologists had access to basic clinical data available in the referral text, typically length of disease, Gleason score and PSA levels. Lesions with acetate uptake visually exceeding the background activity were considered positive. The uptakes were quantified by measurements of the highest standardised uptake value - maximum pixel activity in correlation to body weight and the injected dose, grams per millilitre (standardised uptake value (SUV_max_)). All visually positive uptakes were measured in box regions of interest (ROIs) with PET VCAR software (AW 4.5, General Electric, Pewaukee, WI, USA) to determine SUV_max_. The mediastinal background was measured in reference ROIs. The ROIs were delineated with a threshold of 42% of maximum signal intensity [17,18].

The morphological criteria for suspected lymph node metastases were round shape, short-axis diameter exceeding 10 mm, lack of fat-containing hilus and visually increased contrast enhancement compared to normal lymph nodes.

Other suspected metastatic sites were skeletal and hepatic lesions. The morphological criteria for suspected bone metastasis on CT were sclerotic, lytic or mixed lesions with destruction of cortical bone. Characteristics consistent with suspected hepatic metastases on CT were hypo- or hyperattenuating hepatic masses with irregular delineation and/or pathologic pattern of contrast enhancement.

The distinction between low, intermediate and high grade of suspicion of lymph node metastasis was based on the combination of the level of SUV_max_, morphological changes in size and structure and pathological contrast enhancement, according to generally defined and accepted criteria for evaluation of lymph node involvement in CT as well as in ^18^F-FDG PET studies. Low-grade suspicious lesions have been considered non-metastatic. Intermediate- and high-grade suspicious lesions have been evaluated as metastatic. The systematic interpretation of lymph nodes is described in detail in Tables 2 and 3. Depending on the level of SUV_max_, also normal-appearing lymph nodes can be categorised as intermediate grade, and vice versa, morphologically aberrant lymph nodes with just a slight increase in SUV_max_ are categorised as high grade according to Tables 2 and 3. The high grade of suspicion requires both PET and CT changes, whereas in the intermediate group, the level of the SUV_max_ is the critical parameter. Pelvic locoregional lymph nodes and distant paraaortal lymph nodes were evaluated with slightly different criteria than other distant lymph nodes regarding the impact of the level of SUV_max_, in order to reduce the number of false-positive reactive lymph nodes in the thorax and the inguinal regions, where prostate cancer metastases are less likely to occur (Tables 2 and 3). Paraaortal lymph node metastases per definition are considered distant metastases. Our division of lymph nodes into pelvic and paraaortal versus other distant lymph node metastases was due to anatomical reasons - the lymphatic drainage from the prostate makes it more likely to find metastases in the pelvic and paraaortal lymph nodes. Also, low-grade uptake in small mediastinal lymph nodes is a relatively common finding of unclear clinical significance, making these lymph nodes more difficult to interpret. The paraaortal lymph nodes were thus treated with higher degree of suspicion than other distant sites, with regard to likelihood of metastasis.

**Table 2:** **Criteria for low, intermediate, and high grade of suspicion of pelvic/paraaortal lymph node metastasis in PET/CT**

	**SUV**_**max**_ **< mediastinal background**	**SUV**_**max**_ **> mediastinal background**	**Any visually positive uptake on PET**	**Pathological appearance on CT**
Low grade	+			-
Intermediate grade		+		-
High grade			+	+

**Table 3:** **Criteria for low, intermediate, and high grade of suspicion of distant (except for paraaortal) lymph node metastasis in PET/CT**

	**SUV**_**max**_ **<50% higher than mediastinal background**	**SUV**_**max**_ **>50% higher than mediastinal background**	**Any visually positive uptake on PET**	**Pathological appearance on CT**
Low grade	+			-
Intermediate grade		+		-
High grade			+	+

### Statistical analysis

Different risk groups regarding the estimated risk of locoregional lymph node metastases versus the observed risk were compared with Pearson's chi-square test. The association between prostate SUV_max_, PSA and Gleason score, respectively, and the presence of metastatic disease was evaluated with binary logistic regression analysis. Correlations between prostate SUV_max_, PSA and Gleason score were evaluated using linear regression analysis. For more reliable sample sizes, patients were merged into three Gleason score groups: Gleason score 6 to 7, 8 and 9 to 10. The chosen significance level was *p* <0.05. All statistical analyses were executed in IBM SPSS Statistics 21 (SPSS Inc., Chicago, IL, USA).

## Results

### Distribution of positive ^11^C-acetate PET/CT findings

**Figure 1:**
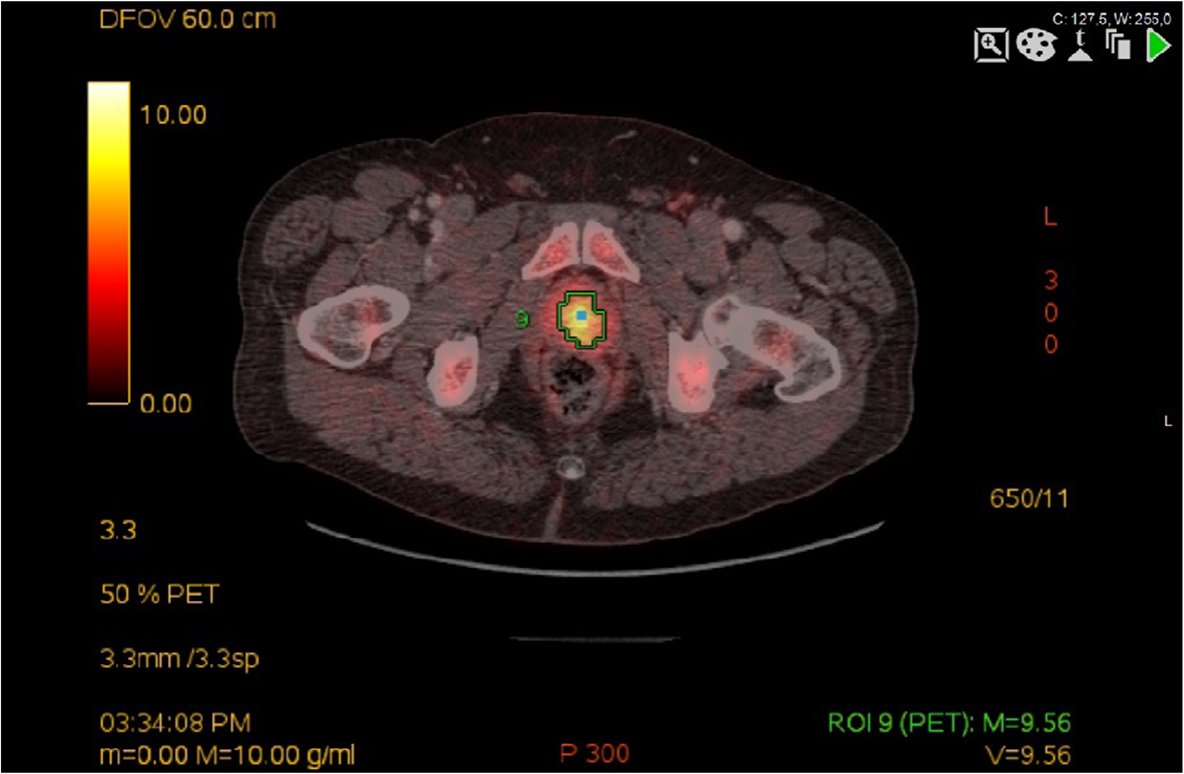
**Increased acetate uptake (SUV**_**max**_ **9.6) of the prostate in patient with prostate cancer.**

**Figure 2:**
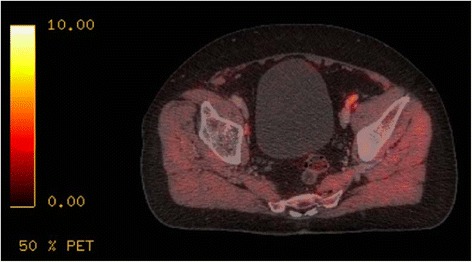
**Increased acetate uptake in suspected iliac lymph node metastasis in a prostate cancer patient.**

All patients showed increased heterogeneous acetate uptake of the prostate (mean SUV_max_ 7.3 g/ml, range 3.3 to 13.8 g/ml); typical appearance is shown in Figure 1. Suspected lymph node metastases were seen in 105 lymph nodes in 42% (21/50) of the patients, most frequently along the external iliac vessels, which was the case in 71% (15/21) of the patients (typical uptake shown in Figure 2). In 45% (47/105) of the positive lymph nodes, only PET was positive, and in the other 55% (58/105), both PET and CT were pathologic. Distribution and characteristics of suspected locoregional lymph node metastases are shown in detail in Table 4.

**Table 4:** **Distribution of total number of intermediate and high-grade suspicious locoregional lymph node uptakes, interpreted as metastatic**

**Locoregional LN localisation**	**High-grade suspicious LN metastases (mean SUV**_**max**_ **g/ml and mean short axis mm)**	**Intermediate suspicious LN metastases (mean SUV**_**max**_ **g/ml)**	**Number of patients (** ***n*** **=21)**	**∑ suspicious LN metastases**
Pararectal	6 (5.4 g/ml, 20 mm)	0	4	6
Obturator	6 (5.2 g/ml, 9.7 mm)	2 (2.9 g/ml)	6	8
External iliac	28 (6.6 g/ml, 14 mm)	36 (3.9 g/ml)	15	64
Internal iliac	11 (6.5 g/ml, 14 mm)	4 (4.7 g/ml)	9	15
Common iliac	7 (4.8 g/ml, 13 mm)	5 (4.3 g/ml)	6	12
∑	58	47	21^a^	105

^a^21 patients with acetate uptakes in multiple locations.

**Figure 3:**
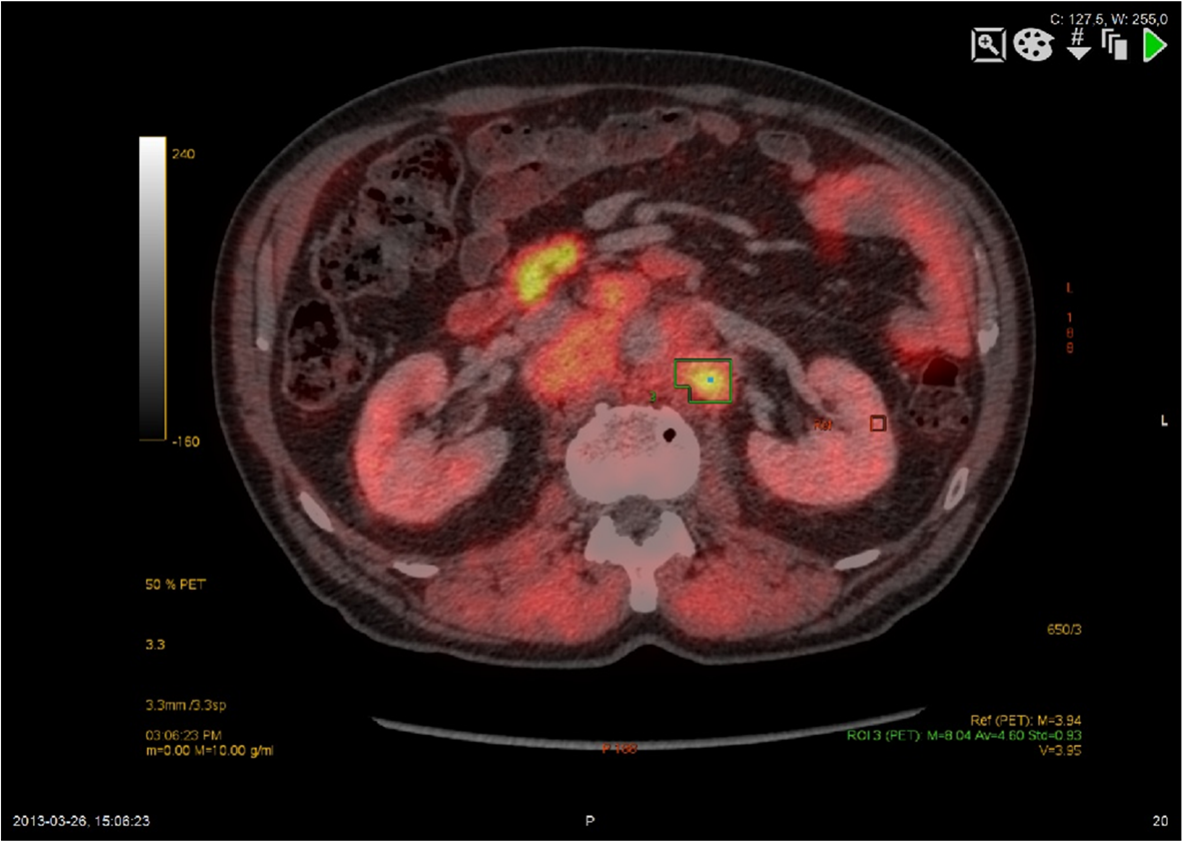
**Increased acetate uptake in pathologically enlarged paraaortal lymph nodes in patient with prostate cancer.**

Previously unknown distant metastases were found in 28% (14/50) of patients: located in paraaortal lymph nodes in six patients, mediastinal lymph nodes in eight and cervical lymph nodes in one patient (example shown in Figure 3). Among the 14 patients with suspected distant metastases were four with skeletal metastases. Unexpected incidental findings in two patients were biopsy-verified synchronous renal cell carcinoma and hepatocellular carcinoma. Distribution of suspected distant metastases and incidental findings are shown in detail in Table 5.

**Table 5:** **Distribution of suspected distant metastases and incidental findings**

**Localisation of suspected pathology**	**Number of PET + lesions**	**Number of CT + lesions**	**CT appearance**	**Pathology unrelated to prostate cancer**	**Number of patients**	**∑ distant metastases**
Paraaortal LN	6 (mean SUV_max_ 5.8 g/ml)	6 (mean short axis 13 mm)	Oval-rounded shape		6	6 (4 solitary, 2 conglomerates)
Mediastinal/hilar LN	8 (mean SUV_max_ 3.7 g/ml)	8 (mean short axis 10 mm)	Oval-rounded shape		8	8 (6 solitary, 2 conglomerates)
Axillary LN	1 (SUV_max_ 9.0 g/ml)	1 (short axis 10 mm)	Rounded shape		1	1
Cervical LN	2 (mean SUV_max_ 5.6 g/ml)	2 (mean short axis 10 mm)	Rounded shape		1	2 (2 conglomerates)
Skeletal	20 (mean SUV_max_ 7.1 g/ml)	20	Sclerotic		4	20
Liver	1 (SUV_max_ 14.3 g/ml)	1	Hypoattenuating mass	HCC	1	-
Kidney	1 (SUV_max_ 14.2 g/ml)	1	Heterogeneous mass	RCC	1	-
∑	39	39			22^a^	37

^a^Six patients with multiple sites, in total 14 with suspected distant metastases, and two with incidental findings. HCC, hepatocellular carcinoma; RCC, renal cell carcinoma.

Low-grade mediastinal lymph node uptake (SUV_max_ range 2.3 to 5.8 g/ml) with low suspicion of lymph node metastasis was seen in 22% (11/50) of the patients with 22 nodal uptakes in total. In 55% (6/11) of these patients, only non-specific mediastinal lymph node uptake was seen and 45% (5/11) of the patients had both non-specific and suspected metastatic mediastinal lymph node uptakes.

### Association between ^11^C-acetate PET/CT findings and the estimated risk of locoregional lymph node metastases

Patients were divided into three groups according to estimated risk of pelvic lymph node metastases, <15% (*n* = 12), 15 to 45% (*n* =24) and >45% (*n* = 13), respectively. The observed proportions of pelvic lymph node metastases in the three groups were 33, 33 and 69%, respectively. The Pearson chi-square test displayed that there was no significant association between the observed proportion and the estimated nomogram risk of pelvic lymph node metastases in the three original groups (*p* = 0.08). Merging the first and the second groups, those with ≤45% estimated risk had positive pelvic lymph nodes in 33% (12/36), and those with >45% risk had positive pelvic lymph nodes in 69% (9/13), and this association proved to be significant (*p* <0.05).

The overall estimated mean risk of pelvic lymph node metastases was 32%, while the observed proportion of suspected pelvic lymph node metastases was 43% (21/49). Twelve patients had an estimated risk <15%. All of these patients had ^11^C-acetate-positive lesions in the prostate and 3/12 patients (25%) showed suspected pelvic lymph node involvement, two with intermediate and one with high suspicion of metastasis.

### Prognostic value of prostate SUV_max_

Binary logistic regression analysis proved prostate SUV_max_ to be higher in patients with suspected pelvic lymph node spread (odds ratio (OR) 1.45, *p* <0.05). Neither prostate SUV_max_, PSA or Gleason score was associated with the presence of suspected distant metastases, nor were PSA or Gleason score associated with suspected pelvic lymph node spread.

There was a significant positive correlation between prostate SUV_max_ and PSA (Pearson correlation coefficient 0.35, *p* <0.05), but not with Gleason score. PSA and Gleason score were negatively correlated (−0.33, *p* <0.05).

### Impact of ^11^C-acetate PET/CT findings on treatment strategy

In 43% (20/47) of the patients, treatment strategy was altered due to ^11^C-acetate PET/CT findings (Table 6). Eleven of these had an estimated risk of pelvic lymph node metastases ≤45% and the remaining nine >45%, with treatment changes as indicated in Table 7. In 19% (9/47) of the patients, suspected pelvic lymph node metastases (SUV_max_ 2.6 to 11.0 g/ml, short axis 5 to 16 mm) were treated with additional radiotherapy boost (mean additional dose 17 Gy, range 15 to 26 Gy). The nine patients that received additional radiotherapy boost against suspected pelvic lymph node metastases were fully informed of the non-conventional approach in this treatment, and all chose to go forward with it. The selected lymph nodes were irradiated according to the proposed Radiotherapy Oncology Group (RTOG) lymph node template. Lymph nodes were only boosted if they were within this field. Otherwise, the patients were considered to have distant metastases. The disease was far more extensive than expected, and therapeutic options were no longer curative in 21% (11/47) of the patients. The findings were confirmed with conventional examinations such as bone scintigraphy, and occasionally with biopsy, according to clinical routine before changing therapy to non-curative. Patients that were switched to palliative treatment received androgen deprivation therapy except for one patient who declined because of a diagnosis of previously unknown synchronous hepatocellular carcinoma accidentally detected on ^11^C-acetate PET/CT. Three out of the eleven patients also got radiotherapy to the prostate to palliate local symptoms. In 1/11 patients, radiotherapy treatment was cancelled due to comorbidity.

**Table 6:** ^**11**^ **C-acetate PET/CT findings resulting in altered treatment strategy**

**Patient number**	**Localisation pelvic LN metastases**	**Localisation distant metastases**	**Synchronous finding**	**Curative → additional RT**	**Curative → non-curative RT**
1	Iliac int/ext	Paraaortal LN	-	-	Palliative RT
2	Iliac ext	Skeletal	-	-	No RT
3	Iliac int/ext	-	-	RT boost 16 Gy	-
4	Iliac int/ext		-	RT boost 16 Gy	-
5	Iliac ext	-	-	RT boost 16 Gy	-
6	Iliac com		-	RT boost 16 Gy	-
7	Iliac int/ext	-	-	RT boost 16 Gy	-
8	Obt, iliac int/ext/com		-	RT boost 15 Gy	-
9	-	-	HCC with paraaortal LN	-	No RT
10	Iliac ext		-	RT boost 20 Gy	-
11	Obt, iliac int/ext/com	Paraaortal LN, skeletal	-	-	No RT
12	Pararectal, iliac ext	-	-	RT boost 26 Gy	-
13	Pararectal, obt, iliac int/ext/com	Paraaortal LN	-	-	Palliative RT
14	Iliac int/ext/com	Paraaortal, thoracic, cervical LN	-	-	No RT
15	-	Skeletal	-	-	No RT
16	Iliac ext	-	-	RT boost 16 Gy	-
17	Iliac ext	Skeletal	-	-	No RT
18	-	-	Aortic aneurysm	-	No RT
19	Pararectal, iliac int/ext/com	Paraaortal, thoracic LN	-	-	No RT
20	Pararectal, obt, iliac ext	-	-	-	Palliative RT
∑	17	8	2	9	11

HCC, hepatocellular carcinoma; LN, lymph nodes; Obt, obturator; RT, radiotherapy.

**Table 7:** **Treatment change due to** ^**11**^ **C-acetate PET/CT findings in estimated risk groups according to Cagiannos nomogram**

**Estimated risk**	**Addition of RT boost to pelvic LN**	**Change to no/palliative RT**
≤45% (*n* =11)	4	7
>45% (*n* =9)	5	4

LN, lymph nodes.

The remaining 57% (27/47) were treated with standard radiotherapy as initially planned. Standard radiotherapy of the prostate gland did not change due to ^11^C-acetate PET/CT results.

## Discussion

There was a statistically significant association between the observed proportions of ^11^C-acetate PET/CT findings suggestive of pelvic lymph node metastases in merged estimated risk groups ≤45 and >45% according to the established Cagiannos nomogram, which supports the value of ^11^C-acetate PET/CT in pelvic lymph node staging of primary prostate cancer. The lack of a statistically significant result in the original three risk groups can be explained by inadequate power of the study since the number of patients in each risk group is low. Surprisingly, patients with previously estimated low (<15%) risk of pelvic lymph node metastases were found to have PET/CT-positive lymph nodes in 25%. The overall observed proportion of positive PET/CT findings suggestive of pelvic lymph node metastases was higher than the estimated risk, suggesting either that ^11^C-acetate PET/CT is superior to the clinical prediction tool or that there is a high rate of false-positive findings. It is possible that the inclusion criteria were such that this resulted in higher numbers, and there may be a selection bias especially since ^11^C-acetate PET/CT was a new method at the time and is still used only in cases with risk of pelvic lymph node involvement and where curative radiotherapy is the intended treatment. That may explain why our results are not comparable with other studies based on low- and intermediate-risk groups, but it does not explain the difference in the observed proportion of suspected pelvic lymph node metastases versus the expected risk in the Cagiannos nomogram. Since the patients in our study were treated with radiotherapy, there was no possibility to obtain histopathological confirmation of the suspected lymph node metastases. Haseebuddin et al. have shown a sensitivity of 68% and specificity of 78% of ^11^C-acetate PET/CT for detecting pelvic lymph node metastases [15], but apart from their study, little is published on suspected lymph node metastases with this method. Another possible explanation for the discordance could be that the Cagiannos nomogram underestimates the risk of lymph node metastasis, as has been previously suggested by Walz et al. in 2012 [19]. Schiavina et al. showed in their study on ^11^C-choline PET/CT in intermediate-risk and high-risk prostate cancer that the specificity and accuracy of PET/CT was better than that of the Kattan and Briganti nomograms, although the results were not statistically significant [20]. Altogether, our conclusion is that the higher proportion of ^11^C-acetate-positive pelvic lymph nodes is not merely an artefact. The distinction between metastatic and non-metastatic unspecific findings in this study was based on the combination of increased SUV_max_, morphological changes in size and structure and pathological contrast enhancement, i.e., the established variables for characterising a lesion as benign or malignant. In prostate cancer, however, some data indicate that pelvic lymph nodes may be enlarged not only due to metastatic disease but also because of associated hyperplastic or regressive alterations [21]. On the other hand, it is also well known that normal-sized lymph nodes can harbour metastases.

The relatively common low intense acetate uptake in otherwise normal-appearing mediastinal lymph nodes is considered non-specific, but the mechanism and possible prognostic features remain unclear. The presence of non-specific mediastinal lymph nodes did not influence therapy, and this finding will be followed clinically. Mediastinal lymphadenopathy in prostate cancer is described in several case reports in the literature as a rare symptom of advanced disease [22–24]. This needs further evaluation, especially with the contradictory findings from a post-mortem study on 176 cases describing a distribution of lymph node metastases from prostate cancer to the paraaortal regions most frequently, followed by the external iliac and tracheobronchial regions [25].

Higher prostate SUV_max_ correlates in our study with the presence of suspected pelvic lymph node metastases, in contrast to earlier studies by Kato et al. concluding that prostate SUV measurements in dynamic ^11^C-acetate PET in known normal prostate and benign prostatic hyperplasia overlap significantly with those for known prostate cancer [26]. The reason for this may be the selection of mainly high-risk prostate cancer patients in our material.

The weaknesses of this study are that two indirect methods to predict metastatic disease in prostate cancer patients are compared and, as before mentioned, that the sample population is relatively small. In future research, the need for histopathologic confirmation is obvious, although one recent study by Haseebuddin et al. shows that the presence of ^11^C-acetate PET/CT-positive pelvic lymph nodes independently predicts treatment failure, despite negative histopathologic findings [15]. Furthermore, there may be a risk of selection bias as mentioned above, but also in the low-risk group (<15%) in this material, since they have been submitted for ^11^C-acetate PET/CT despite their low risk of pelvic lymph node metastases and may have some other characteristic in common, of which we are unaware.

^11^C-acetate is a relatively new tracer for prostate cancer staging with molecular properties reflecting the pattern of lipid metabolism. It could potentially be used for other slow growing cancers apart from prostate cancer such as highly differentiated hepatocellular carcinoma (HCC), renal cell carcinoma (RCC), bladder carcinoma and brain tumours [27,28]. In our material of 50 patients, we found one histology-verified RCC and one HCC, thus supporting previous studies.

The impact of ^11^C-acetate PET/CT on treatment strategy is high and in line with a previous report from Kjölhede et al. where 20% of patients examined with combined ^18^F-choline PET/CT and ^18^F-FDG PET/CT had their treatment plans altered [29]. This result also complies with several other studies on ^18^F-FDG PET/CT in various kinds of cancer with changes in treatment in 21 to 62% of the patients [30–32]. In our study, patients with limited pelvic nodal disease received integrated radiotherapy boost to the pelvic lymph nodes. This is a strategy that has been explored and found feasible regarding toxicity and clinical outcome [5].

Our results indicate that ^11^C-acetate PET/CT might be of value in pelvic lymph node staging in previously untreated high-risk prostate cancer patients, but these results should be interpreted with caution especially since the number of patients was low and histopathological confirmation was absent. However, the prognosis in patients with high-risk prostate cancer is poor, there is no optional treatment, and the risk of negative side effects is low, which makes the possible benefit of added therapy greater than the possible harm. The reason for excluding patients from curative treatment has been unequivocal metastatic disease taken into account the specificity and sensitivity issue. In our ongoing prospective study, survival as well as biochemical response will be assessed.

## Conclusions

^11^C-acetate PET/CT for staging of prostate cancer influences treatment strategy in a substantial way and the use of ^11^C-acetate PET/CT seems to be of value in clinical practice for previously untreated high-risk patients, although the method needs further validation. The overall observed proportion of ^11^C-acetate PET/CT-positive pelvic lymph nodes is higher than the estimated pre-treatment nomogram risk, suggesting the nomogram might underestimate the risk. Prostate SUV_max_ is positively correlated to the presence of suspected pelvic lymph node metastases in ^11^C-acetate PET/CT and to PSA. Further long-term studies are needed to evaluate the impact of ^11^C-acetate PET/CT findings on patient outcome and survival.

## Abbreviations

CECT: contrast-enhanced computed tomography
FDG: fluoro-deoxy-glucose
HCC: hepatocellular carcinoma
i.v.: intravenous
LN: lymph node
OR: odds ratio
PET/CT: positron emission tomography/computed tomography
PSA: prostate-specific antigen
RCC: renal cell carcinoma
ROI: region of interest
RT: radiotherapy
SUV: standardised uptake value
